# Associations of follicle-stimulating hormone and luteinizing hormone with metabolic syndrome during the menopausal transition from the National Health and Nutrition Examination Survey

**DOI:** 10.3389/fendo.2023.1034934

**Published:** 2023-02-09

**Authors:** Yongjie Chen, Caihong Wang, Boran Sun, Bingyi Wang, Xinlin Lu, Bei Gao, Ye Cao, Jiangtao Zhou, Xuewei Liu

**Affiliations:** Department of Epidemiology and Statistics, School of Public Health, Tianjin Medical University, Tianjin, China

**Keywords:** luteinizing hormone, metabolic syndrome, metabolic syndrome severity score, menopausal transition, follicle-stimulating hormone

## Abstract

**Background:**

The increased risk of metabolic syndrome (MetS) during the menopausal transition might partly attribute to the changes in follicle-stimulating hormone (FSH) and luteinizing hormone (LH). However, few studies were conducted to examine the associations of FSH and LH concentrations with MetS at the full range of reproductive aging, especially in the US population. The aim of this study is to examine the associations of FSH, LH, and LH/FSH ratio with the risk of MetS and severity score in the US women.

**Methods:**

Data were derived from the National Health and Nutrition Examination Survey. Women aged from 35 to 60 years were eligible. MetS was defined as having at least 3 of the following: a waist circumference ≥ 88 cm, a triglycerides level ≥ 150 mg/dL, a high density lipoprotein < 50 mg/dL, a systolic blood pressure ≥ 130 mm Hg or a diastolic blood pressure ≥ 85 mm Hg or taking hypertension medications, or a fasting plasma glucose level ≥100 mg/dL or taking diabetes medications. The MetS severity score was calculated according to race/ethnicity- specific equation.

**Results:**

There were 3,831 women included in this study. Increases in serum FSH and LH levels per 1 SD were separately linked to a 22.6% (*OR*: 0.774; *95% CI*: 0.646, 0.929; and *P*= 0.006) and 18.5% (*OR*: 0.815; *95% CI*: 0.690, 0.962; and *P*= 0.006) lower risk of MetS only in postmenopausal women. Meanwhile, increases in serum FSH and LH levels per 1SD were associated with a decrease of -0.157 (*95% CI* :-2.967, -2.034) and -0.078 (*95% CI*: -2.688, -1.806) MetS severity score in perimenopausal women and -0.195 (*95% CI*: -2.192, -1.023) and -0.098 (*95% CI*:-1.884, -0.733) in postmenopausal women. However, LH/FSH ratio had no connections with the risk of MetS and MetS severity score across the menopausal transition.

**Conclusions:**

Elevated serum FSH and LH levels, but not LH/FSH ratio, were associated with a lower risk of MetS and MetS severity score, especially in postmenopausal women. Therefore, serum FSH and LH levels might be efficient predictors for screening and identifying women at risk of MetS across the menopausal transition.

## Introduction

Metabolic syndrome (MetS) is characterized by a combination of central obesity, high blood pressure, blood sugar and triglycerides (TG), and low high density lipoprotein (HDL) cholesterol (1). In US, the prevalence of MetS was 34.7% among adults, and remained stable from 2011 to 2016 (2). However, the risk of MetS will considerably increase after shifting into menopause (3). It was declared that the prevalence of MetS will increase to 31.0-55.0% in postmenopausal women, which is not only associated with aging, but also associated with the changes in sex hormones including estrogen, follicle-stimulating hormone (FSH), and luteinizing hormone (LH) during the menopausal transition (3–5). Therefore, identifying the specific factors of MetS across the menopausal transition may contribute to the prevention of MetS.

There were many studies to investigate the associations of estrogen and androgen with MetS (6–8). However, no evidence on the association of estrogen with MetS were found. Furthermore, a different incidence of MetS was found in postmenopausal women with the same level of estrogen (9). Recently, the potential roles of FSH and LH in MetS are increasingly followed with interest. However, to date, few studies were conducted to evaluate the associations of FSH and LH with the risk of MetS. The existent studies limited to a small sample size, postmenopausal women alone, and FSH alone (9–12). Similar data regarding the relationship between FSH and MetS at the full range of reproductive aging are lacking. Furthermore, no study has be conducted to examine the association of FSH concentration with MetS in the US population until now. On the other hand, it is well documented that race/ethnicity significantly affects the incidence of MetS (13–16). Therefore, it is necessary to evaluate the association of FSH with MetS severity score, which can correct the racial/ethnic differences.

In this study, data derived from the National Health and Nutrition Examination Survey (NHANES) were used to examine the associations of FSH, LH, and LH/FSH ratio with the prevalence of MetS and severity score in the US women.

## Materials and methods

### Study design

The NHANES is a repeated cross-sectional survey and aims to assess and supervise the health and nutritional status in the US population. A stratified, multistage random procedure is used to recruit sample from the US population. The major information collected in the NHANES include demographic, socioeconomic, dietary, health-related behaviors, physiological measurements, and laboratory tests. Since 1959, a series of surveys were conducted in different population groups. To meet emerging needs, the NHANES has become a continuous program since 1999. The NHANES III focused on oversampling many groups, including children aged 2 months to 5 years, older adults aged 60 years or over, Mexican-American persons, and non-Hispanic black persons. However, the continuous NHANES focused on oversampling of low-income group, adolescents aged 12-19 years, older adults aged 60 years or over, African Americans, and Mexican Americans. Since data of both serum FSH and LH levels are publicly accessible only in the NHANES III and the continuous NHANES from 1999 to 2002, only the data from the NHANES III and the NHANES from 1999 to 2002 were used in this study. The NHANES III from 1988-1994 randomly recruits 39,695 participants aged 2 months and older. The continuous NHANES from 1999 to 2002 includes the NHANES 1999-2000 and the NHANES 2001-2002, which recruit 9,965 and 11,039 participants of all ages, respectively. The details of the NHANES are available at the website: https://www.cdc.gov/.

### Study population

**Figure 1:**
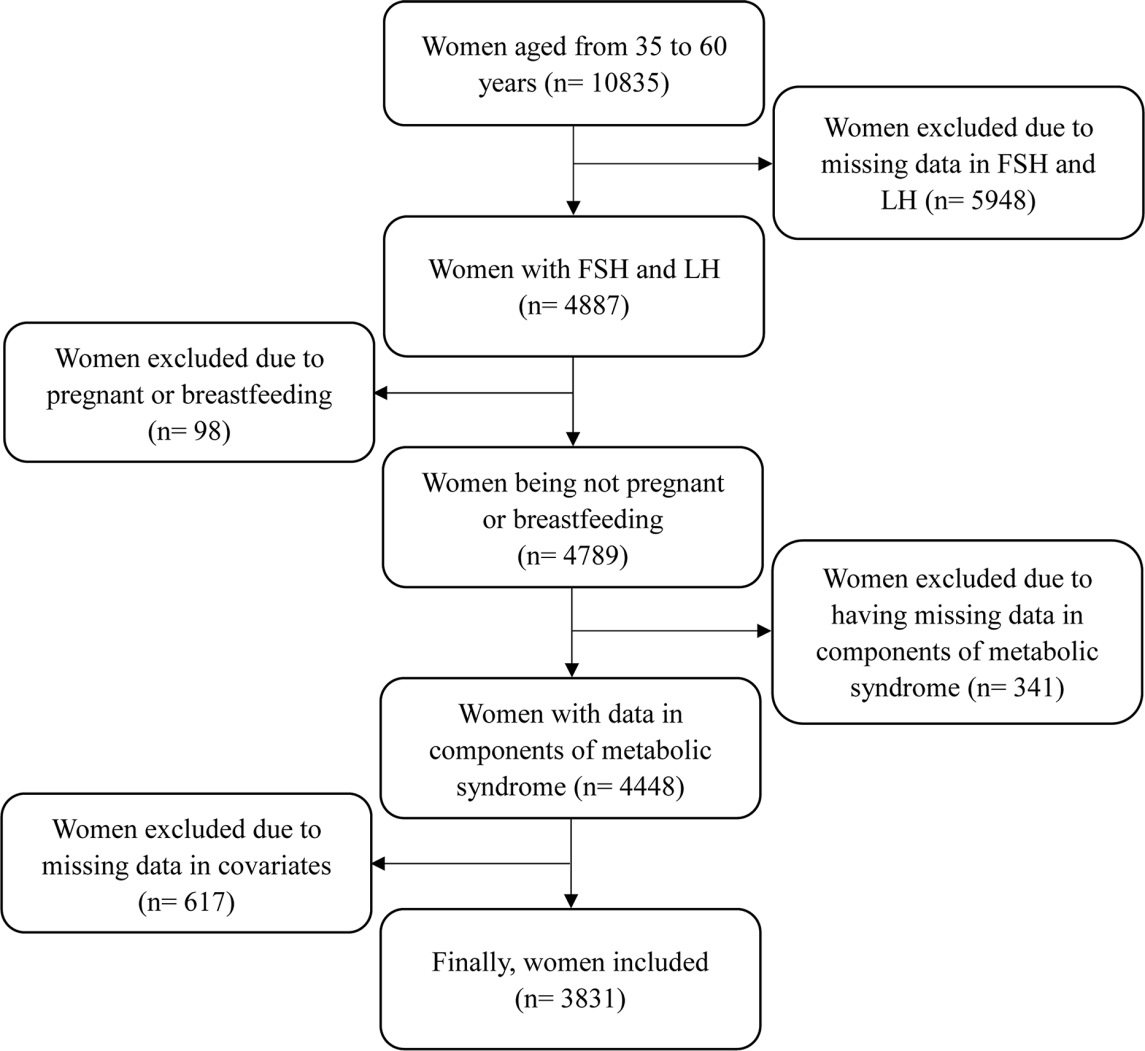
The detailed process of participants included.

Since serum FSH and LH levels were collected only in women aged 35-60 years, this study only focused on women aged from 35 to 60 years. The inclusion criteria included: women having complete data of serum FSH and LH levels; women having complete data of metabolic risk factors, including waist circumference (WC), TG, HDL cholesterol, systolic blood pressure (SBP), diastolic blood pressure (DBP), and fasting plasma glucose levels; and women having complete data of main covariates, such as physical measurements, health-related indicators, and reproductive health indicators. The exclusion criteria included: women being pregnant or breastfeeding at the time of survey; and women having missing data of analyzed variables. The detailed process is shown in **Figure 1**. This study was approved by the NHANES Institutional Review Board (1999-2002: Protocol #98-12). Documented consent was obtained from participants.

### Measurements

Fasting blood samples were collected in the mobile examination center and were used to assay serum FSH and LH levels using the Microparticle Enzyme Immunoassay technology (IMx FSH assay, IMx LH assay, Abbott Laboratories). The inter-assay coefficient of variation (CV) varied from 2.37 to 7.95 for FSH and from 1.65 to 10.1 for LH. The intra-assay CV varied from 3.20 to 4.11 for FSH and from 4.64 to 6.08 for LH. Serum TG levels were assayed using Hitachi Model 917 Multichannel Analyzer. Serum HDL levels were assayed using Hitachi 704 Analyzer. Plasma glucose levels were assayed using Enzyme hexokinase. Standard mercury sphygmomanometer was used to measure DBP and SBP, which were indicated by the first and fifth Korotkoff sounds, respectively. The averages of three measures were used in the final analysis. A question of “Because of your hypertension, have you ever taken prescribed medicine?” was used to identify whether taking hypertension medications. Similarly, a question was used to identify whether taking diabetes medications as follows: Other than during pregnancy, have you ever been told by a doctor or health professional that you have diabetes or sugar diabetes? WC was measured at the midpoint between the lower margin of the least palpable rib and the top of the iliac crest using a stretch-resistant tape.

### Definition of MetS and calculation of MetS severity score

According to the National Cholesterol Education Program’s Adult Treatment Panel III, MetS was defined as having at least 3 of the following in women: a WC ≥ 88 cm, a TG level ≥ 150 mg/dL, a HDL level < 50 mg/dL, a SBP ≥ 130 mm Hg or a DBP ≥ 85 mm Hg or taking hypertension medications, or fasting plasma glucose level ≥100 mg/dL or taking diabetes medications (17).

Since sex and race/ethnicity significantly affect the incidence of MetS, all participants were divided into six subgroups based on sex and race/ethnicity to correct the influences of sex and race/ethnicity. Since this study only involved women, there were three race/ethnicity subgroups (non-Hispanic White, non-Hispanic Black, and Hispanic). For each subgroup, data derived from the NHANES 1999–2010 among adults aged 20-64 years were used to determine the weights of MetS components of WC, TG, HDL cholesterol, SBP, and fasting plasma glucose using the confirmatory factor analysis. Factor loadings from factor analysis were used to generate separately equations for each of three subgroups. The MetS severity score was calculated according to specific equation, which was described previously (18, 19).

### Covariates

Covariates included age, race/ethnicity (non-Hispanic White, non-Hispanic Black, and Hispanic), current smoking (yes or no), current alcohol consumption (yes or no), annual household income, history of heart disease (yes or no), history of stroke (yes or no), parturiency status (yes or no), had a unilateral oophorectomy (yes or no), and use of hormone therapy (yes or no). Hispanic included Mexican-American and other Hispanic. Current smoking status was identified using a question as follows: Do you now smoke cigarettes/pipe/cigars/use snuff/use chewing tobacco? Alcohol consumption included consumption of liquor (such as whiskey or gin), beer, wine, wine coolers, and any other type of alcoholic beverage. Annual household income was classified as < $20,000, $20,000~$45,000, and ≥ $45,000. History of heart disease was identified using a series of questions as follows: Has a doctor or other health professional ever told you that you had congestive heart failure/coronary heart disease/angina/angina pectoris/heart attack? History of stroke was identified using a question as follows: Has a doctor or other health professional ever told you that you had a stroke? Menopausal status was categorized according to the STRAW criteria as follows: premenopausal category included all four premenopausal categories (i.e. −5 to −3), perimenopause category included the two menopausal transition categories (−2 and −1) and one early postmenopausal category (+1a), and postmenopausal category included three postmenopausal categories (+1b, +1c, and +2) (20, 21).

### Statistical analysis

*Kolmogorov-Smirnov test* was used to test for normality of continuous variables. Continuous data with normal distribution are expressed as means ± standard deviations (SDs) and are compared between Non-MetS and MetS groups using *t*-test. Due to the abnormal distribution, serum FSH and LH levels are expressed as *P_50_* (*P_25_*, *P_75_*) and are compared between-group using *Wilcoxon rank sum* test. Categorized variables are expressed as frequencies (percentages) and are compared using *chi-square* test. General linear regression was employed to analyze the associations of serum FSH and LH levels and LH/FSH ratio with MetS severity score by menopausal status. Logistic regression model was used to obtain odd ratios (*ORs*) and 95% confidential intervals (*CIs*) of serum FSH and LH levels and LH/FSH ratio with the prevalence of MetS and each component of MetS by menopausal status. Furthermore, serum FSH and LH levels were further divided into quintiles. In multivariable models, age, race/ethnicity, current smoking, current alcohol consumption, annual household income, history of heart disease, history of stroke, parturiency status, had a unilateral oophorectomy, and use of hormone therapy were adjusted. Furthermore, the sampling weight provided in the NHANES dataset was taken into account to adjust for non-responses bias and over-sampling of certain populations using PROC SURVEYLOGISTIC and PROC SURVEYREG in SAS 9.4. Meanwhile, multivariate Imputation by Chained Equations (*mice*) package in the statistical program R (version 3.5.1) was used to impute missing data of all the variables to investigate the impact of missing data. A sensitivity analysis was conducted to examine the associations of serum FSH and LH levels and LH/FSH ratio with the risk of MetS and MetS severity score using the complete dataset. All analyses were conducted using SAS 9.4 (SAS Institute Inc., Cary, NC, USA.). A two-tailed *P*≤ 0.05 was considered to be statistically significant.

## Results

### Characteristics of all women

There were 3,831 women included in this study. There were 728 premenopausal women, 1,502 perimenopausal women, and 1,601 postmenopausal women. The prevalence of MetS was 34.01%. The average of age was 45.94 ± 7.45 years. The medians of serum FSH and LH levels were 11.00 (5.60, 50.76) IU/L and 8.60 (3.50, 22.98) IU/L, respectively. The mean of LH/FSH ratio was 0.73 ± 0.84. The mean of MetS severity score was 0.15 ± 1.28. **Table 1** shows the comparisons between non-MetS and MetS groups in all characteristics. Significant differences were observed in all characteristics, except current smoking (*P*= 0.089).

**Table 1:** Characteristics of all participants.

Characteristics	All participants (n=3,831)	Non-MetS (n=2,528)	MetS (n=1,303)	*P*
Age (mean ± SD, years)^※^	45.94 ± 7.45	44.91 ± 7.22	47.94 ± 7.49	<0.001
WC (mean ± SD, cm) ^※^	93.93 ± 15.41	88.42 ± 13.24	104.61 ± 13.60	<0.001
Race/Ethnicity (n (%))^※^				<0.001
Non-Hispanic White	1701 (44.40)	1221 (48.30)	480 (36.84)	
Non-Hispanic Black	997 (26.02)	648 (25.63)	349 (26.78)	
Hispanic	1133 (29.57)	659 (26.07)	474 (36.38)	
Current smoking (n (%))^※^				0.089
No	2926 (76.38)	1952 (77.22)	974 (74.75)	
Yes	905 (23.62)	576 (22.78)	329 (25.25)	
Current alcohol consumption (n (%))^※^				<0.001
No	2536 (66.20)	1612 (63.77)	924 (70.91)	
Yes	1295 (33.80)	916 (36.23)	379 (29.09)	
Annual household income (n (%))^※^				<0.001
< $20,000	1207 (31.51)	671 (26.54)	536 (41.14)	
$20,000~$45,000	1311 (34.22)	860 (34.02)	451 (34.61)	
≥$45,000	1313 (34.27)	997 (39.44)	316 (24.25)	
History of heart disease (n (%))^※^				<0.001
No	3769 (98.38)	2509 (99.25)	1260 (96.70)	
Yes	62 (1.62)	19 (0.75)	43 (3.30)	
History of stroke (n (%))^※^				0.002
No	3782 (98.72)	2506 (99.13)	1276 (97.93)	
Yes	49 (1.28)	22 (0.87)	27 (2.07)	
Parturiency status (n (%))^※^				0.006
No	293 (7.65)	215 (8.50)	78 (5.99)	
Yes	3538 (92.35)	2313 (91.50)	1225 (94.01)	
Had a unilateral oophorectomy (n (%))^※^				<0.001
No	3210 (83.79)	2182 (86.31)	1028 (78.89)	
Yes	621 (16.21)	346 (13.69)	275 (21.11)	
Use of hormone therapy (n (%))^※^				<0.001
No	2953 (77.08)	1993 (78.84)	960 (73.68)	
Yes	878 (22.92)	535 (21.16)	343 (26.32)	
Menopausal status (n (%))^※^				<0.001
Premenopause	728 (19.00)	549 (21.72)	179 (13.74)	
Perimenopause	1502 (39.21)	1091 (43.16)	411 (31.54)	
Postmenopause	1601 (41.79)	888 (35.13)	713 (54.72)	
Serum FSH levels (*P_50_* (*P_25_*, *P_75_*), IU/L) **^§^**	11.00 (5.60, 50.76)	9.12 (5.30, 48.37)	20.80 (6.45, 53.40)	<0.001
Serum LH levels (*P_50_* (*P_25_*, *P_75_*), IU/L) **^§^**	8.60 (3.50, 22.98)	6.96 (3.27, 22.62)	11.70 (4.20, 23.11)	<0.001
LH/FSH (mean ± SD)^※^	0.73 ± 0.84	0.76 ± 0.85	0.67 ± 0.81	0.003
SBP (mean ± SD, mmHg)^※^	122.76 ± 18.26	118.13 ± 15.96	131.75 ± 19.07	<0.001
DBP (mean ± SD, mmHg)^※^	76.34 ± 10.91	74.54 ± 9.88	79.83 ± 11.91	<0.001
Fasting plasma glucose (mean ± SD, mg/dL)^※^	98.45 ± 39.00	88.68 ± 15.35	117.31 ± 58.71	<0.001
TG (mean ± SD, mg/dL)^※^	131.98 ± 95.10	98.55 ± 49.60	196.84 ± 124.29	<0.001
HDL (mean ± SD, mg/dL)^※^	54.70 ± 15.47	59.51 ± 15.07	45.37 ± 11.45	<0.001
MetS severity score	0.15 ± 1.28	-0.42 ± 0.67	1.27 ± 1.42	<0.001

MetS, metabolic syndrome; FSH, follicle-stimulating hormone; LH, luteinizing hormone; WC, waist circumference; SBP, systolic blood pressure; DBP, diastolic blood pressure; HDL, high density lipoprotein; TG, triglyceride.

^※^These variables were analyzed using t-test.

^※^These variables were analyzed using chi-square.

**^§^** These variables were analyzed using Wilcoxon rank sum test.

### Associations of FSH, LH, and LH/FSH ratio with components of MetS

As shown in **Table 2**, increase in serum FSH and LH levels per 1 SD were linked to a lower risk of central obesity in pre-, peri-, and postmenopausal women. For other components of MetS, increase in serum FSH and LH levels per 1 SD were negatively associated with elevated TG, reduced HDL, and elevated plasma glucose only in postmenopausal women but not in pre- and perimenopausal women. Meanwhile, an increase in serum FSH levels per 1 SD was negatively associated with blood pressure in postmenopausal women (*P*= 0.012), while an increase in LH levels was negatively associated with blood pressure in perimenopausal women (*P*= 0.025). However, LH/FSH ratio was not associated with all components of MetS across the menopausal transition.

**Table 2:** The associations of FSH, LH, and LH/FSH with components of metabolic syndrome^※^.

Response variables	Predictors	Premenopausal women (n=728)		Perimenopausal women (n=1502)		Postmenopausal women (n=1601)^#^	
		*OR (95% CI)*	*P*	*OR (95% CI)*	*P*	*OR (95% CI)*	*P*
Central obesity	FSH	0.529 (0.317,0.884)	0.015	0.710 (0.583,0.863)	0.001	0.697 (0.620,0.784)	<0.001
	LH	0.738 (0.572,0.952)	0.019	0.814 (0.693,0.956)	0.012	0.830 (0.747,0.922)	0.001
	LH/FSH	0.908 (0.764,1.080)	0.276	1.058 (0.970,1.153)	0.204	0.997 (0.777,1.278)	0.979
Elevated TG	FSH	1.259 (0.721,2.199)	0.418	1.018 (0.821,1.262)	0.869	0.758 (0.672,0.855)	<0.001
	LH	1.027 (0.756,1.395)	0.864	0.906 (0.746,1.099)	0.316	0.843 (0.754,0.941)	0.003
	LH/FSH	0.949 (0.752,1.197)	0.658	1.077 (0.994,1.168)	0.071	1.167 (0.918,1.484)	0.207
Reduced HDL	FSH	0.702 (0.401,1.230)	0.216	0.899 (0.736,1.098)	0.295	0.815 (0.727,0.914)	0.001
	LH	0.905 (0.700,1.171)	0.449	0.912 (0.773,1.077)	0.278	0.845 (0.759,0.942)	0.002
	LH/FSH	0.919 (0.769,1.098)	0.35	0.989 (0.916,1.068)	0.782	0.972 (0.767,1.232)	0.814
Elevated blood pressure	FSH	1.060 (0.638,1.761)	0.823	0.870 (0.715,1.060)	0.167	0.865 (0.773,0.968)	0.012
	LH	1.103 (0.845,1.439)	0.472	0.820 (0.690,0.976)	0.025	0.994 (0.897,1.101)	0.903
	LH/FSH	1.071 (0.880,1.302)	0.495	0.957 (0.853,1.073)	0.449	1.237 (0.975,1.568)	0.08
Elevated plasma glucose	FSH	1.004 (0.518,1.945)	0.991	0.955 (0.764,1.194)	0.687	0.884 (0.781,1.001)	0.051
	LH	0.781 (0.501,1.219)	0.277	0.884 (0.722,1.081)	0.228	0.866 (0.769,0.975)	0.018
	LH/FSH	0.877 (0.657,1.172)	0.377	0.849 (0.708,1.017)	0.076	0.810 (0.603,1.089)	0.164

FSH: follicle-stimulating hormone; LH: luteinizing hormone; TG: triglyceride; HDL: high density lipoprotein

^※^In all models, age, race/ethnicity, current smoking, current alcohol consumption, annual household income, history of heart disease, history of stroke, parturiency status, had a unilateral oophorectomy, and use of hormone therapy were adjusted.

^#^In postmenopausal women, years since menopause was additionally adjusted.

### Associations of FSH, LH, and LH/FSH ratio with the risk of MetS

The associations of FSH, LH, and LH/FSH ratio with MetS are displayed in **Table 3**. An increase in serum FSH levels was linked to a 22.6% lower risk of MetS only in postmenopausal women (*OR*: 0.774; *95% CI*: 0.646, 0.929; and *P*= 0.006), but not in premenopausal women (*P*= 0.524) and perimenopausal women (*P*= 0.355). Similarly, an increase in serum LH levels was related to an 18.5% lower risk of MetS only in postmenopausal women (*OR*: 0.815; *95% CI*: 0.690, 0.962; and *P*= 0.016), but not in premenopausal women (*P*= 0.469) and perimenopausal women (*P*= 0.426). However, no significant relationship between LH/FSH ratio and the prevalence of MetS was observed in pre-, peri-, and postmenopausal women (*P*= 0.710, 0.911, and 0.801).

**Table 3:** The associations of FSH, LH, and LH/FSH with the risk of metabolic syndrome^※^.

Models	Premenopausal women (n=728)		Perimenopausal women (n=1,502)		Postmenopausal women (n=1,601)^#^	
	*OR( 95% CI)*	*P*	*OR (95% CI)*	*P*	*OR (95% CI)*	*P*
Model 1						
FSH	0.795 (0.393,1.610)	0.524	0.861 (0.627,1.182)	0.355	0.774 (0.646,0.929)	0.006
Model 2						
LH	1.144 (0.794,1.649)	0.469	0.900 (0.695,1.166)	0.426	0.815 (0.69,0.962)	0.016
Model 3						
LH/FSH	1.053(0.801,1.384)	0.71	1.009 (0.866,1.176)	0.911	0.964 (0.722,1.286)	0.801

FSH, follicle-stimulating hormone; LH, luteinizing hormone.

^※^In all models, age, race/ethnicity, current smoking, current alcohol consumption, annual household income, history of heart disease, history of stroke, parturiency status, had a unilateral oophorectomy, and use of hormone therapy were adjusted.

^#^In postmenopausal women, years since menopause was additionally adjusted.

**Figure 2:**
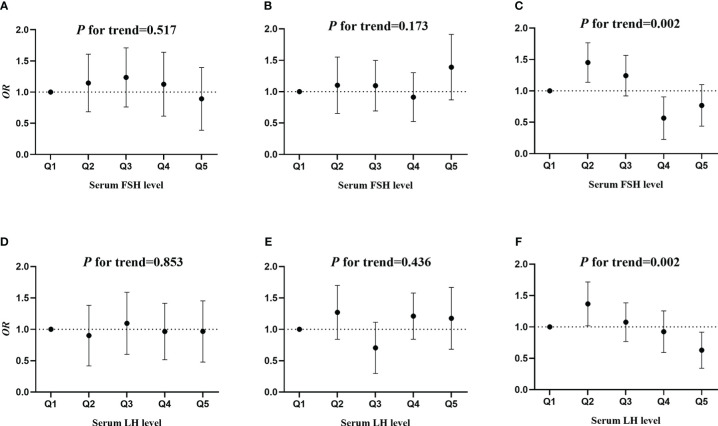
The associations of serum FSH and LH quintiles with the risk of MetS. **(A, D)** premenopausal women, **(B, E)** perimenopausal women, and **(C, F)** postmenopausal women.

The associations of serum FSH and LH quintiles with the prevalence of MetS are shown in **Figure 2**. No significant associations of serum FSH quintiles with the risk of MetS were observed both in pre- and perimenopausal women (*P* for trend= 0.517 and 0.173) as shown in **Figures 2A, B**. However, in postmenopausal women, compared to the lowest quintile of FSH, the second quintile of FSH was linked to a higher risk of MetS (*OR*: 1.452; *95% CI*: 1.138, 1.766; and *P*= 0.020), but the fourth quintile of FSH was related to a lower risk of MetS (*OR*: 0.566; *95% CI*: 0.228, 0.904; and *P*= 0.001) as shown in **Figure 2C**. Similarly, there were no significant associations of serum LH quintiles with MetS both in pre- and perimenopausal women (*P* for trend= 0.853 and 0.436) as shown in **Figures 2D, E**. However, a significant association of serum LH quintile with MetS was observed in postmenopausal women (*P* for trend= 0.002), and a negative association of fifth quintile of LH with the risk of MetS was observed (*OR*: 0.629; *95% CI*: 0.342, 0.916; and *P*= 0.002) as shown in **Figure 2F**.

### Associations of FSH, LH, and LH/FSH ratio with MetS severity score

**Table 4** shows the associations of FSH, LH, and LH/FSH ratio with MetS severity score. No significant association of serum FSH levels per 1 SD change with MetS severity score was observed in premenopausal women (*b*: -0.064; *95% CI*: -1.697,-0.228; and *P*= 0.569). However, an increase in serum FSH levels was associated with a decrease of -0.157 (*95% CI*: -2.967,-2.034) in MetS severity score in perimenopausal women and -0.195 (*95% CI*: -2.192,-1.023) in postmenopausal women. Similarly, an increase in serum LH levels was linked to a decrease of -0.078 (*95% CI*: -2.688,-1.806) in MetS severity score in perimenopausal women and -0.098 (*95% CI*: -1.884,-0.733) in postmenopausal women. However, LH/FSH ratio has no concern with MetS severity score in pre-, peri-, and postmenopausal women (*P*= 0.095, 0.208, and 0.843).

**Table 4:** The associations of FSH, LH, and LH/FSH with metabolic syndrome severity score^※^.

Models	Premenopausal women (n=728)		Perimenopausal women (n=1,502)		Postmenopausal women (n=1,601)^#^	
	*b(95% CI)*	*P*	*b(95% CI)*	*P*	*b(95% CI)*	*P*
Model 1						
FSH	-0.064 (-1.697,-0.228)	0.569	-0.157 (-2.967,-2.034)	<0.001	-0.195 (-2.192,-1.023)	<0.001
Model 2						
LH	-0.044 (-1.551,-0.271)	0.34	-0.078 (-2.688,-1.806)	0.006	-0.098 (-1.884,-0.733)	<0.001
Model 3						
LH/FSH	-0.056 (-1.399,-0.127)	0.095	0.028 (-2.444,-1.629)	0.208	-0.012 (-1.774,-0.600)	0.843

FSH, follicle-stimulating hormone; LH, luteinizing hormone.

^※^In all models, age, current smoking, current alcohol consumption, annual household income, history of heart disease, history of stroke, parturiency status, had a unilateral oophorectomy, and use of hormone therapy were adjusted.

^#^In postmenopausal women, years since menopause was additionally adjusted.

**Figure 3:**
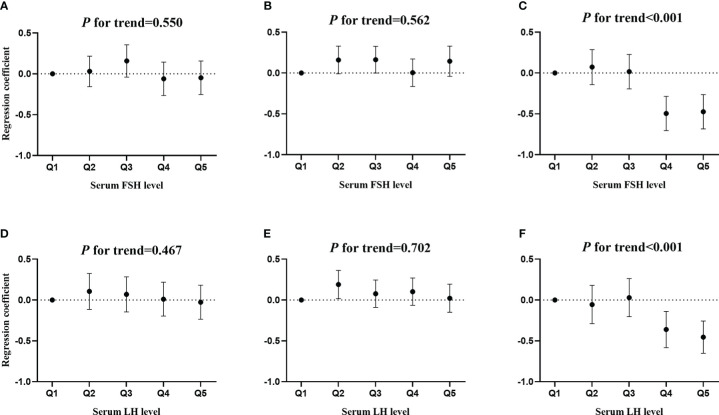
The associations of serum FSH and LH quintiles with MetS severity score. **(A, D)** premenopausal women, **(B, E)** perimenopausal women, and **(C, F)** postmenopausal women.

**Figure 3** shows the associations of serum FSH and LH quintiles with MetS severity score. There was significant associations of serum FSH quintile with MetS severity score only in postmenopausal women (*P* for trend< 0.001, as shown in **Figure 3C**). Compared to the lowest quintile of FSH, only the fourth (*b*: -0.496; *95% CI*: -0.706, -0.286; and *P* < 0.001) and fifth (*b*: -0.474; *95% CI*: -0.684, -0.264; and *P* < 0.001) quintiles of FSH were associated with a lower Met severity score. Similarly, a significant association of serum LH quintiles with MetS severity score was observed in postmenopausal women (**Figure 3F**). Furthermore, only the fourth (*b*: -0.361; *95% CI*: -0.584, -0.139; and *P*= 0.002) and fifth (*b*: -0.454; *95% CI*: -0.652, -0.256; and *P* < 0.001) quintiles of LH were associated with a lower Met severity score in postmenopausal women.

### Sensitivity analysis

The associations of serum FSH and LH levels with the risk of MetS and MetS severity score using multiple imputation dataset are displayed in **Supplemental Tables S1**, **S2**. An increase in serum FSH levels was associated with a 25.0% (*OR*: 0.750; *95% CI*: 0.965, 0.535; and *P*= 0.011) lower risk of MetS in perimenopausal women and 21.9% (*OR*: 0.781; *95% CI*: 0.916, 0.646; and *P*= 0.003) in postmenopausal women. Meanwhile, an increase in serum LH levels was associated with an 18.7% (*OR*: 0.813; *95% CI*: 0.675, 0.951; and *P*= 0.013) lower risk of MetS in postmenopausal women. Similarly, both serum FSH and LH levels were negatively associated with MetS severity score in peri- and postmenopausal women, which was consistent with the main results.

## Discussion

In this study, a cross-sectional study was designed to assess the associations of serum FSH and LH levels and LH/FSH ratio with the risk of MetS and MetS severity score in women across the menopausal transition. Elevated serum FSH and LH levels had connections with a lower risk of MetS only in postmenopausal women. Meanwhile, there were negative associations of serum FSH and LH levels with MetS severity score in peri- and postmenopausal women, but not in premenopausal women. However, LH/FSH ratio had no connections with the risk of MetS and MetS severity score across the menopausal transition.

In this study, it was found that elevated serum FSH levels were linked to a decreased risk of MetS in postmenopausal women, which was in line with previous studies (9, 11, 12, 22, 23). Since MetS is a cluster of cardiometabolic risk factors including central obesity, blood pressure, blood sugar and TG, and HDL cholesterol, the association of serum FSH levels with MetS was mostly dependent of the associations of serum FSH levels with the components of MetS (24, 25). In this study, higher FSH levels were related to lower WC, TG, blood pressure, and plasma glucose in postmenopausal women. As a result, it was rational that there was a negative link between serum FSH levels and MetS. However, a few previous studies declared that FSH can promote lipid biosynthesis and visceral fat accumulation, and was positively related to fat mass (26–29). It seems that the results of this study were inconsistent with previous studies. Therefore, further longitudinal study should be well designed to confirm the relationship between serum FSH levels and the risk of MetS.

Furthermore, previous studies found a negative relationship between serum FSH levels and diabetes in postmenopausal women, which supported the finding of this study and might partly attribute to adiposity, insulin resistance, and inflammatory factors caused by FSH in the pathogenesis of diabetes (30, 31). On the other hand, it was reported that lower FSH levels were related to a higher blood pressure, which was consistent with the finding of this study (9, 11).

Another finding of this study was that serum FSH levels have concern with MetS only in postmenopausal women, but not in pre- and perimenopausal women. It was speculated that cardiovascular metabolic factors are protected by estrogen before menopause but not during the menopausal transition and beyond (32–34). On the other hand, the association of serum FSH levels with MetS severity score showed that there were significant associations in both peri- and postmenopausal women, but not in premenopausal women. These different associations might attribute to the impact of race/ethnicity on sex hormones and MetS. It was well documented that sex hormones trajectories and the incidence of MetS were not uniform across different race/ethnicity subgroups over the menopausal transition (13, 35). Since MetS severity score was calculated according to race/ethnicity- specific subgroups, there was no impact of race/ethnicity on the associations of serum FSH and LH quintiles with MetS severity score. Meanwhile, this might explain the differences between the associations of serum FSH and LH levels with the risk of MetS and the associations of serum FSH and LH levels with MetS severity score.

Furthermore, similar to FSH, serum LH levels were also negatively related to the risk of MetS only in postmenopausal women. FSH and LH act by pituitary–ovarian axis and change synchronously during the menopausal transition. Furthermore, both serum FSH and LH levels are regulated by the steroid hormones through feedback loop mechanisms (36). Therefore, it was reasonable that there were similar associations of serum FSH and LH levels with MetS. In this study, no significant relationships between LH/FSH ratio and the risk of MetS and MetS severity score were observed across the menopausal transition. Therefore, LH/FSH ratio might not be a potentially effective predictor of MetS in women. The underlying mechanisms on the associations of serum FSH and LH levels and LH/FSH ratio with the risk of MetS and MetS severity score need be further investigated in the future.

### Strengths and limitations

This study had some strengths. Firstly, this study provided a comprehensive investigation on the associations of serum FSH levels with MetS and MetS severity score at the full range of reproductive aging. Secondly, this study firstly examined the associations of serum LH levels and LH/FSH ratio with the risk of MetS and MetS severity score across the menopausal transition. Therefore, this study will provide additional evidence for the prevention of MetS across the menopausal transition. However, there were also some limitations in this study. Firstly, a cross-sectional design was used in this study. It is difficult to establish the temporal associations of serum FSH and LH levels with MetS. Secondly, due to lack of data of serum estrogen levels in women aged 35-60 years in the NHANES, serum estrogen levels were not adjusted in this study. Thirdly, the underlying mechanisms on the associations of serum FSH and LH levels with MetS were not fully explained in this study. Fourthly, fasting blood samples were collected to assay serum FSH and LH levels in a random time but not a specific time. Therefore, there might be fluctuation in serum FSH and LH levels during different phases of menstrual cycle in premenopausal women.

In conclusion, elevated serum FSH and LH levels were linked to a lower risk of MetS only in postmenopausal women, but not in pre- and perimenopausal women. Meanwhile, negative associations of serum FSH and LH levels with MetS severity score were observed in peri- and postmenopausal women. However, LH/FSH ratio was not associated with the risk of MetS and MetS severity score across the menopausal transition. Identifying the associations of FSH and LH with the risk of MetS may help identify women at risk of MetS according to serum FSH and LH levels and contribute to the precision prevention of MetS.

## Data availability statement

The datasets presented in this study can be found in online repositories. The names of the repository/repositories and accession number(s) can be found below: https://www.cdc.gov/nchs/index.htm.

## Ethics statement

The studies involving human participants were reviewed and approved by NHANES Institutional Review Board (1999-2002: Protocol #98-12). The patients/participants provided their written informed consent to participate in this study.

## Author contributions

CW and BS contributed to writing the original draft. BW and XiL contributed to review and editing the draft. BG, YeC, JZ, and XuL contributed to formal analysis and results interpretation. YoC contributed to study design and conceptualization. All authors contributed to the article and approved the submitted version.
