# Supplementary material for: Associations of follicle-stimulating hormone and luteinizing hormone with metabolic syndrome during the menopausal transition from the National Health and Nutrition Examination Survey
Source: Front Endocrinol (Lausanne). 2023 Feb 9;14:1034934. doi: 10.3389/fendo.2023.1034934 (PMC9947143; doi:10.3389/fendo.2023.1034934)
Supplement: Supplementary file 1 [file Table_1.docx]

Table S1 The associations of FSH, LH, and LH/FSH with the risk of metabolic syndrome using multiple imputation dataset^※^

| Models | Premenopausal women  (n= 2,034) | | Perimenopausal women  (n= 4,294) | | Postmenopausal women  (n= 4,397) ^#^ | |
| --- | --- | --- | --- | --- | --- | --- |
|  | *OR(95% CI)* | *P* | *OR(95% CI)* | *P* | *OR(95% CI)* | *P* |
| **Model 1** |  |  |  |  |  |  |
| FSH | 0.664 (1.256,0.072) | 0.196 | 0.750(0.965,0.535) | 0.011 | 0.781(0.916,0.646) | 0.003 |
| **Model 2** |  |  |  |  |  |  |
| LH | 1.030 (0.737,1.323) | 0.846 | 0.823 (0.587,1.060) | 0.141 | 0.813(0.675,0.951) | 0.013 |
| **Model 3** |  |  |  |  |  |  |
| LH/FSH | 1.007 (0.807,1.207) | 0.947 | 0.967(0.822,1.112) | 0.655 | 0.997(0.755,1.239) | 0.981 |

FSH: follicle-stimulating hormone; LH: luteinizing hormone

^※^In all models, age, race/ethnicity, current smoking, current alcohol consumption, annual household income, history of heart disease, history of stroke, parturiency status, had a unilateral oophorectomy, and use of hormone therapy were adjusted.

^#^ In postmenopausal women, years since menopause was additionally adjusted.

Table S2 The associations of FSH, LH, and LH/FSH with metabolic syndrome severity score using multiple imputation dataset^※^

| Models | Premenopausal women  (n= 2,034) | | Perimenopausal women  (n= 4,294) | | Postmenopausal women  (n= 4,397) ^#^ | |
| --- | --- | --- | --- | --- | --- | --- |
|  | *b(95% CI)* | *P* | *b(95% CI)* | *P* | *b(95% CI)* | *P* |
| **Model 1** |  |  |  |  |  |  |
| FSH | -0.212 (-0.513,0.089) | 0.147 | -0.178 (-0.290,-0.066) | 0.003 | -0.172 (-0.232,-0.113) | <0.001 |
| **Model 2** |  |  |  |  |  |  |
| LH | 0.002(-0.161,0.165) | 0.979 | -0.094 (-0.173,-0.015) | 0.022 | -0.121 (-0.182,-0.061) | <0.001 |
| **Model 3** |  |  |  |  |  |  |
| LH/FSH | -0.022 (-0.142,0.097) | 0.656 | -0.010 (-0.057,0.037) | 0.658 | -0.017 (-0.160,0.125) | 0.777 |

FSH: follicle-stimulating hormone; LH: luteinizing hormone

^※^In all models, age, current smoking, current alcohol consumption, annual household income, history of heart disease, history of stroke, parturiency status, had a unilateral oophorectomy, and use of hormone therapy were adjusted.

^#^ In postmenopausal women, years since menopause was additionally adjusted.
